# Comparative efficacy and cognitive function of magnetic seizure therapy vs. electroconvulsive therapy for major depressive disorder: a systematic review and meta-analysis

**DOI:** 10.1038/s41398-021-01560-y

**Published:** 2021-08-21

**Authors:** Miao Chen, Xuhui Yang, Chaojie Liu, Jianying Li, Xiao Wang, Chunxia Yang, Xiaodong Hu, Jianhong Li, Juan Zhao, Xinrong Li, Yong Xu, Sha Liu

**Affiliations:** 1 Department of Psychiatry, First Hospital/First Clinical Medical College of Shanxi Medical University, 030001 Taiyuan, China; 2 Shanxi Key Laboratory of Artificial Intelligence Assisted Diagnosis and Treatment for Mental Disorders, First Hospital of Shanxi Medical University, 030001 Taiyuan, China; 3 College of Humanities and Social Sciences, Shanxi Medical University, 030001 Taiyuan, China

**Keywords:** Depression, Psychiatric disorders

## Abstract

Magnetic seizure therapy (MST) has established efficacy in the treatment of depression and a growing evidence base in the treatment of depression. We conducted the first systematic review and meta-analysis of the efficacy of MST in anti-depressive treatment and its impact on cognitive function (INPLASY registration number: INPLASY202170061). We searched for controlled trials published in English between 1 January 2001 to 31 December 2020 in PubMed, EMBASE, Cochrane Library, Web of Science, and PsycINFO databases. The evaluation process strictly followed the Cochrane bias risk assessment tool into the literature, and Meta-analysis was performed according to the Cochrane System Reviewer’s Manual. Data from a total of 285 patients from 10 studies were retained in the quantitative synthesis. The results showed no significant difference between MST and ECT in the antidepressant effect (SDM −0.13 [−0.78;0.52]). Compared with ECT, MST showed shorter recovery time (MD −5.67 [−9.75; −1.60]) and reorientation time (MD −14.67 [−27.96; −1.41]); and MST showed less cognitive impairment on the immediate recall of words (SDM 0.80 [0.35;1.25]), delayed recall of words (SDM 0.99 [0.01;0.74]), visual-spatial immediate memory (SDM 0.51 [0.20;0.83]), visual-spatial delayed memory (*SDM* 0.57 [0.11;1.02]), and the verbal fluency (*SDM* 0.51 [0.20;0.83]). Our evidence-based study is the first meta-analysis on the efficacy of MST in anti-depressive treatment and its effect on cognitive function. It showed that the curative effect of MST in anti-depressive treatment is equivalent to that of ECT. Besides, depressive patients with MST benefit more from cognitive function compared with ECT.

## Introduction

According to the World Health Organization, depression is the leading cause of disability, affecting more than 300 million people worldwide [1]. Approximately 33% of patients experienced relief of their depressive symptoms after an antidepressant trial. However, general treatment such as medication and psychotherapy were failed in ~30% of the patients [2]. At present, Electroconvulsive therapy (ECT) is widely considered to be one of the effective antidepressant treatments, especially for major depressive disorder (MDD) [3], with remission rates ranging from 40% to 70% [4]. However, previous studies have found that ECT impairs patients’ cognitive functions, especially memory function [5]. Therefore, due to fear and concerns about cognitive impairment caused by ECT, the wide range of clinical use of ECT is limited [6].

Magnetic seizure therapy (MST) is an emerging physical therapy method for antidepressant treatment in recent years [7, 8]. Studies proved that MST treatment of MDD could not only significantly relieve depression symptoms and maintain good cognitive status, but its antidepressant effect also may be related to the local metabolic changes of the bilateral frontal cortex of MDD [9, 10]. The MST technology uses transcranial magnetic stimulation (TMS) to continuously stimulate the cerebral cortex with high-frequency strong pulsed magnetic fields. It induces convulsive seizures by exciting the local cortex. It combines the antidepressant effect of ECT and the minimal adverse reaction of TMS [5]. Compared with TMS, MST has a higher magnetic field frequency and a more substantial output voltage, which is better for treating severe mental disorders. Compared with ECT, MST can more accurately induce currents in the cerebral cortex’s surface space and selectively stimulate the local cerebral cortex without affecting the deep brain nuclei [11]. In recent years, more and more studies have continuously confirmed that MST has better efficacy and more overall benefits than ECT in the treatment of MDD [12–14].

Regarding the side effects of MST and ECT treatment, Dwork AJ applied MST and ECT to animal models and found no histological damage to the brain. Subsequently, it was also found that induced seizures do not cause structural damage to the brain in a rigorous treatment model of human ECT and MST [15]. It was found that there were headaches, nausea, vomiting, and muscle soreness after ECT treatment [14], but no serious adverse events occurred after MST treatment, and the symptoms of headache and nausea were lighter than those after ECT treatment [16, 17].

Besides, Compared with ECT, MST has fewer side effects, including reducing postoperative faster recovery and reorientation [14, 18]. Kayser et al. [14] have found that MST has a shorter seizure time than ECT, and a shorter duration of multi-peak phase can also be a faster indicator of reorientation observed after MST. Previous studies have shown that this measurement method can predict memory side effects [19, 20]. This profile might be one of the most important advantages of MST over ECT, the disorientation after ECT is explained as short retrograde forgetting.

Cognitive impairment is a core symptom of MDD [21], and patients with depression have moderate deficits in executive function, memory, and attention [22]. the current conclusions on the efficacy and cognition of MST in treating depression remain inconsistent. Although many clinical studies have found that MST can effectively alleviate depressive symptoms without adverse cognitive side effects, there are also some opposite findings [11, 23]. Secondly, the current impact of MST and ECT on cognitive function is still controversial. In particular, which cognitive dimensions are specifically affected [9, 24, 25]. Thirdly, current MST-related clinical studies are small-sample studies, even some of which are case reports. A single small-sample study leads to low consistency in overall results. At present, there is no systematic review and meta-analysis of MST. Therefore, it is necessary to make an overall evaluation based on the evidence of the efficacy and cognitive function of MST.

This study aims to conduct a systematic review and use meta-analysis to quantitatively analyze the antidepressant efficacy of MST and ECT and its impact on cognitive function and provide a valuable reference for further promoting MST in clinical practice.

## Materials and methods

### Study selection

This systematic review and meta-analysis followed procedures from the INPLASY (https://inplasy.com/). The review protocol was pre-registered in INPLASY(INPLASY202170061). This review was carried in accordance with the PRISMA [26]. We conducted a systematic literature search in PubMed, Embase, Cochrane Library, PsyclNFO databases and Web of Science databases, using both keywords and MeSH terms for articles published date for searched paper from the database ranged from 1 January 2001 to 31 December 2020. The keywords included are depression, depressive disorder, major depressive disorder, MDD, magnetic seizure therapy, and MST. After retrieval, two researchers read all qualified studies separately, and conducted a preliminary screening through all qualified studies abstract, and reached an agreement after discussion when there were objections; then read the full text and performed two searches on the relevant references of the qualified studies obtained from the preliminary retrieval, and obtained the appropriate inclusion Standard documentation.

### Eligibility criteria

Inclusion criteria: (1) subjects: subjects were depressed patients with a disease diagnosis that met the Diagnostic and Statistical Manual of Mental Disorders (DSM) diagnostic criteria for depressive episodes (including major depression diagnostic criteria and bipolar disorder diagnostic criteria) or the patient has moderate or higher depression; (2) age ≥18 years; (3) intervention measures: magnetic seizure therapy; (4) control group: electroconvulsive therapy; and (5) clinical symptom indicators: the main outcome indicator is the total score of the depression scale and the score of each dimension of the neuropsychological test; Secondary indicators are reorientation time and recovery time. Exclusion criteria: (1) the full text or original data is missing(e.g., meeting abstracts); (2) high-risk bias: studies were assessed using the Cochrane Risk of Bias Assessment Tool and excluded if four or more of them were high risk; (3) repeated published literature; (4) animal experiments, review literature, case studies; (5) unclear intervention methods, A study without a control group; and (6) There is a large difference in the observed indicators, and the effect size cannot be combined (Outcome indicators do not match, such as the definition of research indicators is different). According to the inclusion and exclusion criteria, the abstract and full text of the literature were screened.

### Data extraction

Import the retrieved documents into EndnoteX9. The following information was extracted from all qualified studies by two researchers independently: author, publication year, study design, sample size, average age, duration of illness, clinical indicators, ECT parameters, MST parameters, and duration for treatments (Tables 1 and 2). We extracted test score with standard deviation (SD), sample size, and *P* values for effective size (ES) generation.

**Table 1:** Characteristics of included studies.

Author	Study design	Diagnostic criteria	Sample size		Sex ratio (male/female)		Average age (year)		Duration of illness (year)		Rating scale	Cognitive assessment scale
			Test group (MST)	Control group (ECT)	Test group (MST)	Control group (ECT)	Test group (MST)	Control group (ECT)	Test group (MST)	Control group (ECT)		
Zhang et al. [12]	Par	DSM-IV HAMD ≥ 17	18	27	2/16	5/22	29.00 (8.32)	32.78 (8.84)	3.77 (3.77)	4.47 (5.42)	HAMD-17	RBANS
El-Deeb et al. [32]	Par	DSM-IV-TR	30	15RULECT 15BLECT	13/17	17/13	23.93 (8.17)	BL25.47 (9.33) RUL30.2 (10.52)	7.73 (5.55)	BL6 (5.41) RUL5.73 (4.03)	HAMD-21	–
Paul et al. [10]	Par	HAMD > 18, DSM-IV	18	19	10/8	6/13	44.6 (14.8)	47.2 (16.1)	22.7 (14.3)	27.6 (14.4)	HAMD	–
Atluri et al. [31]	Par	DSM-IV	24	22	12/12	8/14	42(13.4)	46.8 (15.8)	20.3 (13.7)	19 (12)	HAMD-17	–
Kayser et al. [28]	Par	DSM-IV	10	10	7/3	6/4	45 (14)	55 (12)	–	–	HRSD28	–
Polster et al. [13]	Par	DSM-IV	10	10	7/3	4/6	43.7 (11)	54.7 (13)	4.1 (4)	3.1 (3)	–	Memory
Kayser et al. [29]	Cro	DSM-IV	7	–	5/2	–	43.43 (5.59)	–	6.29 (6.04)	–	HRSD28	–
Kayser et al. [14]	Par	DSM-IV, HDRS-28 ≥ 20	10	10	4/6	3/7	48.80 (8.35)	52.8 (11.43)	6.01 (10.42)	3.5 (4.12)	HRSD28	Neuropsychological assessment
Whit et al. [30]	Par	HAMD	10	10	–	-–	48 (4)	49 (6)	–	–	HAMD	–
Lisanby et al. [16]	Cro	DSM-IV	10		–	–	46.7 (10.0)	–	–	–	HRSD-24	Neuropsychological assessment

*Par* parallel, *Cro* crossover, *DSM-IV* the Diagnostic and Statistical Manual of Mental Disorders-IV, *HAMD* Hamilton Depression Rating Scale for Depression, *HAMD-21* Hamilton Depression Scale 21 items, *HAMD-17* Hamilton Depression Scale 17 items, *HRSD-24* Hamilton Rating Scale for Depression 24 items, *HRSD28* Hamilton Rating Scale for Depression 28 items, *RBANS* Repeated Battery for the Assessment of Neuropsychological Status, *RULECT* right unilateral ECT, *BLECT* bilateral ECT.

**Table 2:** Specific parameters of ECT and MST.

Author	ECT parameters			MST parameters				Frequency
	Equipment	Stimulus setting	Stimulation pulse width	Equipment	Location	Stimulation intensity	Duration	
Zhang et al. [12]	SometicsThymatron	Bifrontal electrode	–	Circular coil, 130 mm in diameter,	International standard 10–20 EEG system to establish coil center position, P3.P4 center	100HZ100%	10 s	6
El-Deeb et al. [32]	Thymatron IV, (Somatics LLC, USA).	RULECT and BLECT	0.5 ms	Magstim Theta device	Vertex stimullation	100HZ100%	10 s	2
Fitzgerald et al. [10]	Thymatron IV	RULECT d’Elia electrode	1.0 ms	MagventureA/S, double cone MST coil (13 cm in diameter)	–	100HZ100%	up to 10 s	15
Atluri et al. [31]	Spectrum 5000Q ECT device	RULECT and BLECT	–	Mag Pro MST uses two coils (Mag Venture)	International Standard 10–20 EEG system	–	–	−24
Kayser et al. [28]	Thymatron IV	RULECT	0.5 ms	MagProMST (MagVentureA/S, Farum, denmark) double coil	International Standard 10–20 EEG system, Vertex Stimulation	100HZ100%	8 s	8–12
Polster et al. [13]	AThymatron IV, Somatics LLC	RULECT	0.5 ms	MagPro (MagVentureA/S, Farum, denmark), One double coil and two separate 13 cm wide coils	The center of the coil is placed at the vertex	100HZ100%	5–8 s	10–12
Kayser et al. [29]	Thymatron IV, Somatics LLC	RULECT and BLECT	0.5 ms	MagProMST (MagVentureA/S denmark), Double coil, 13 cm in diameter	Electroencephalogram position of international standard 10–20 EEG system and vertex stimulation on Cz	100HZ100%	Increase by 1 s each time	–
Kayser et al. [14]	Thymatron IV (Somatics LLC, USA & Canada)	RULECT	0.5 ms	MagProMST (MagVentureA/S, Denmark), double coils, 13 cm in diameter	The center of the coil is placed at the vertex	100HZ100%	6 s	12
Whit et al. [30]	Spectrum 5000Q ECT device	–	0.5 ms	Magstim (Magstim Co., Ltd., Wales, United Kingdom)	–	50HZ100%	8 s	10–12
Lisanby et al. [16]	Spectrum 5000Q ECT device	RULECT and BLECT	0.5 ms	MST is managed using an improved magnetic stimulator and 16 booster modules (Magstim, Whitfield, Wales, UK)	RUL position F6, midline frontal position Fz, and vertex position Cz of international standard 10–20 EEG system	40–60HZ100%	0.5s–8s	–

*RULECT* right unilateral ECT, *BLECT* bilateral ECT.

The main outcome indicator is the total score of the depression scale and the score of each dimension of the neuropsychological test, such as the immediate recall of words, delayed recall of words, visual-spatial immediate memory, visual-spatial delayed memory, verbal fluency, and other dimensions; secondary indicators are reorientation time and recovery time (The reorientation time is defined until the patient correctly remembers the time of four of the following five items: name, weeks, birthdate, age, and place; The recovery time is defined as the time until the patient opens his eyes and breathes independently).

### Quality assessment

Evaluation of literature quality by two researchers. If there is any ambiguity, a third researcher will be asked to evaluate. Using the Cochrane Quality Evaluation Scale to assess the quality of included studies: (1) Whether to assign randomly; (2) Whether to describe the allocation method; (3) Whether to blinding of participants and personnel; (4) Whether to blinding of outcome assessment; (5) Whether the outcome dates are complete, includes missed visit and dropout data and reasons; (6) Whether the results are reported selectively. For example, the outcome index report is not complete enough to be included in the analysis; and (7) Whether there are other risks of bias (In addition to the above factors, are there other factors that cause bias, such as treatment standards, adverse events, etc) [27]. The level of risk of bias is expressed as “low risk” and “high risk” respectively, and “unclear” is used when the article has insufficient information. If each type of bias is low risk, a single study is considered to have low risk of bias and high quality; If the risk of one or more types of bias is unknown, it is considered that the risk of bias of a single study is unknown and the quality is medium; If four or more types of bias are at high risk, then a single study is considered to have high risk of bias and low quality.

The publication bias was evaluated by using Stata15.1 to make a funnel chart and a biased score. The absence of obvious publication bias was suggested when the data in a funnel plot were distributed roughly symmetrically and vice versa. Egger’s linear regression was used to test the symmetry of the funnel plot, and a probability value of *P* < 0.05 was considered suggestive of significant asymmetry.

### Statistical analysis

Using Review Manager 5.3 software to assess the risk bias of the included all qualified studies. And the size of heterogeneity of the studies was assessed by combining *I*^2^ statistic and *P* values: *I*^2 ^≥ 50% or *P* < 0.05 indicates high heterogeneity, Sensitivity analysis is used to find the reasons for the heterogeneity, the random-effects model is used for meta-analysis; I^2 ^< 50% or *P* > 0.1, indicating that the research is homogeneous, and the fixed effects model is used.

Meta-analysis was carried out according to the Cochrane System Reviewer’s Manual. Observation indicators included in this study are continuous variables, Since the scores of each test are continuous variables and the scale version used in each document is different, the standardized mean difference (SMD) is selected as the combined effect size; Mean Difference (MD) for Reorientation and Recovery Time. The main indicators are the change score of the Hamilton Depression Scale and the score of each dimension of neuropsychological assessment, Secondary indicators are reorientation time and recovery time. The difference is statistically significant with *P* < 0.05.

## Results

### Study selection and study characteristics

**Fig. 1:**
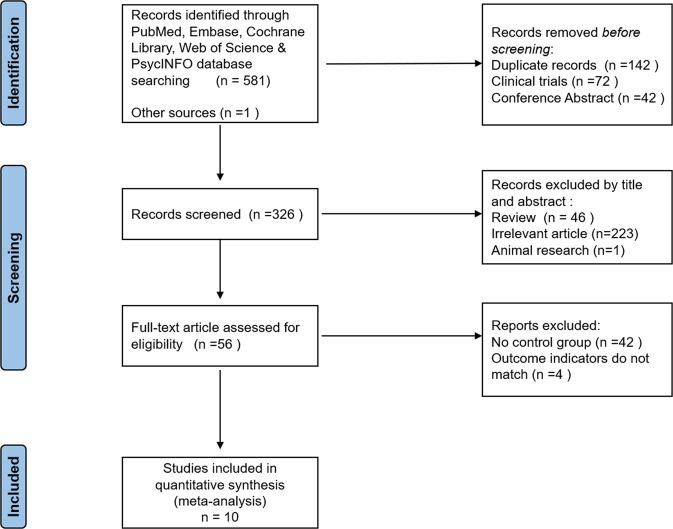
Flow chart of study selection.

Initial screenings identified 581 records from Web of Science, PubMed, Embase, Cochrane Library, and PsycINFO databases. Besides, 1 article was obtained by reading references. After removing duplicate publications, clinical trials and conference abstract, 326 articles were obtained, Due to 46 systematic reviews and meta-analysis articles, 223 irrelevant article, and 1 animal research were excluded, 56 articles were obtained. In total, 270 substandard articles were excluded by reading the titles and abstracts. These records were screened, which led to full-text scrutiny of 56 articles. After carefully reading the 56 articles, 42 articles without a control group, 4 outcome indicators do not meet the inclusion criteria. Finally, 10 articles were included for meta-analysis, including a total of 285 subjects. The process of literature screening is shown in Fig. 1.

### Basic characteristics and risk bias assessment of included literature

Among the 10 articles finally included, 4 articles are randomized controlled trials (RCT) [10, 13, 16, 28], 1 article is a controlled crossover experiment [29], and 5 articles are non-randomized controlled trials [12, 14, 30–32]. Due to the particularity of the intervention treatment, it is difficult to achieve a randomized group study. Ten articles all reported the loss of subjects, and the test results were relatively complete, and there was no document specifically describing the distribution concealment. According to the Cochrane Quality Evaluation Scale, each study had three or more types of low risk and no four or more categories of high risk. Therefore, the 10 included studies have medium bias risks and medium quality. The basic characteristics of the literature are shown in Table 1, the specific parameters of ECT and MST stimulation are shown in Table 2, and the risk assessment of risk bias is shown in eFig.  1.

### The efficacy of antidepressant treatment of MST and its impact on cognition

#### Meta-analysis results of the efficacy of MST antidepressant treatment

**Fig. 2:**
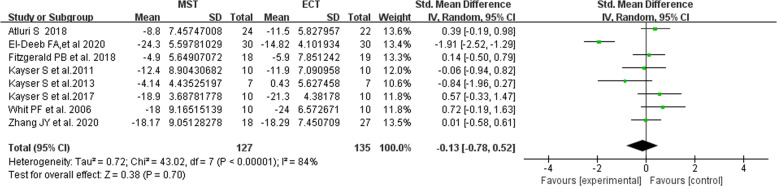
Forest plot of the efficacy of MST and ECT in the treatment of depression.

Eight studies used the HAMD scale change score to evaluate the efficacy of treating depression symptoms, and a total of 262 patients were included [10, 12, 14, 28–32]. It should be noted that, as shown in the Fig. 2, there are differences in scale change score before and after treatment between MST and ECT, but generally speaking, there is no significant difference in the efficacy of MST and ECT in the treatment of depression, and there is a large heterogeneity between studies (eFig. 2).

**Fig. 3:**
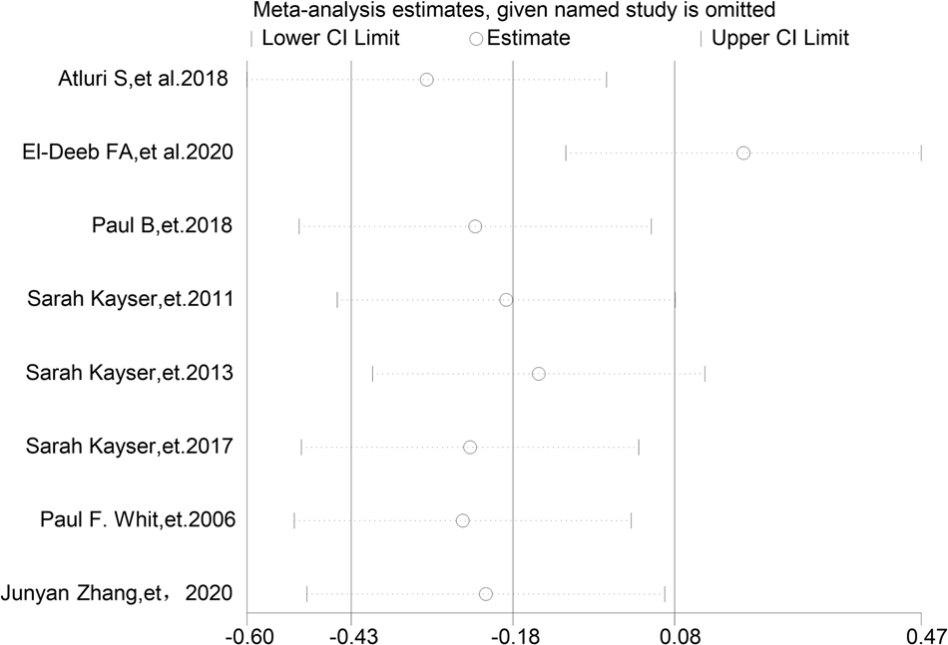
Relative effects of each separate dataset on the pooled effect size for MST and ECT effects on depression.

Due to the high heterogeneity, sensitivity analysis by analyzing the influence of single studies showed that the total pooled effect size was greatly affected by the studies of El-Deeb et al. [32]. Excluding this study produced results that the antidepressant effect of the two groups had non-significant, indicated that the MST and the ECT have the same therapeutic effect (Fig. 3).

#### Results of reorientation and recovery time of MST antidepressant treatment

**Fig. 4:**
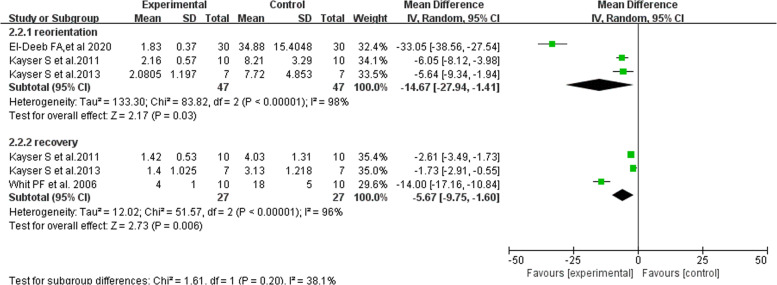
Forest plot of reorientation and recovery time of MST antidepressant treatment.

Three studies measured the patients’ reorientation time treated with MST and ECT, and a total of 94 patients were included [14, 29, 32], and all studies showed that the reorientation time was shorter in the MST group. There was high heterogeneity among the studies, and there was still a significant difference in reorientation time between the two groups after one study was excluded from the sensitivity analysis. There were indicating that reorientation time of MST group was shorter than that of the ECT group (Fig. 4 and eFig. 3).

Three studies measured the recovery time of patients treated with MST and ECT, and a total of 54 patients were included [14, 29, 30], and all studies showed that the recovery time was shorter in the MST group. There was high heterogeneity among the studies, and there was still a significant difference in recovery time between the two groups after one study was excluded from the sensitivity analysis. They were indicating that the recovery time of MST group was shorter than that of the ECT group (Fig. 4 and eFig.3).

#### Results of the impact of MST antidepressant treatment on cognition

**Fig. 5:**
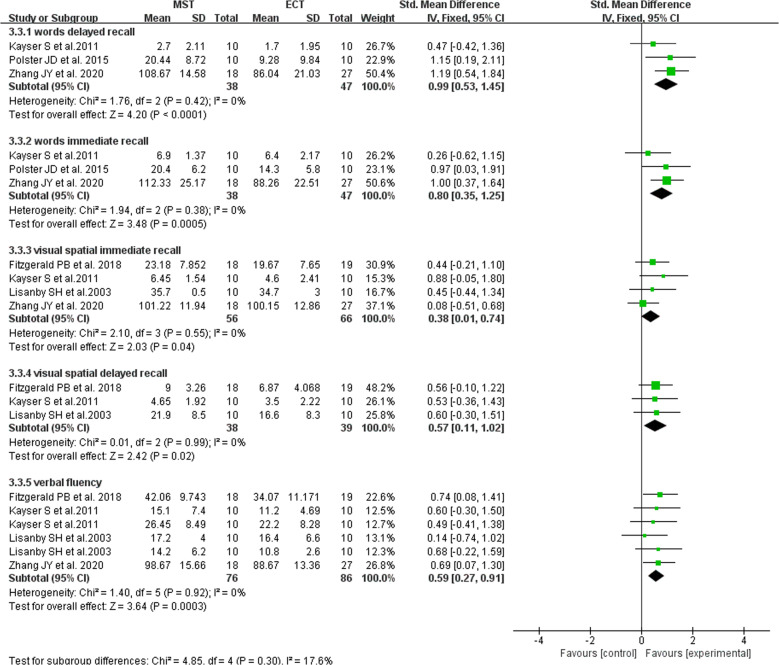
Forest plot of the impact of MST antidepressant treatment on cognition.

Three studies used neuropsychological evaluation to evaluate the efficacy of delayed recall of words in patients with depression, and a total of 85 patients were included [12–14]. There was no heterogeneity between studies, and fixed effect model was used for analysis. The study found that there were significant differences in delayed recall of words between the two groups, and the effect of MST treatment on delayed recall of words was better than that after ECT treatment (Fig. 5).

Three studies used neuropsychological evaluation to evaluate the effect of depression patients on immediate word recall, and a total of 85 patients were included [12–14]. There was no heterogeneity between studies, and fixed effect model was used for analysis. The study found that there were significant differences in the immediate word recall scores between the two groups, and the effect on immediate word recall after MST treatment better than that after ECT treatment (Fig. 5).

Four studies used neuropsychological evaluation to evaluate the efficacy of immediate visual-spatial recall in patients with depression, and a total of 112 patients were included [10, 12, 14, 16]. There was no heterogeneity between studies, and fixed effect model was used for analysis. The study found that there were significant differences in immediate visual-spatial recall between the two groups, and the effect of MST treatment on visual space memory was better than ECT the effect after treatment (Fig. 5).

Three studies used neuropsychological evaluation to evaluate the efficacy of delayed recall of visual-spatial in patients with depression, and a total of 67 patients were included [10, 14, 16]. There was no heterogeneity between studies, and fixed effect model was used for analysis. The study found that there were significant differences in delayed recall of visual-spatial between the two groups, and the effect of MST treatment on visual space delay memory was better than that after ECT treatment (Fig. 5).

Four studies used neuropsychological evaluation to evaluate the efficacy of verbal fluency in patients with depression, and a total of 112 patients were included [10, 12, 14, 16]. There was no heterogeneity between studies, and fixed effect model was used for analysis. The study found that there were significant differences in verbal fluency between the two groups, and the effect of MST treatment on language fluency was better than that after ECT treatment (Fig. 5).

### Publication bias

**Fig. 6:**
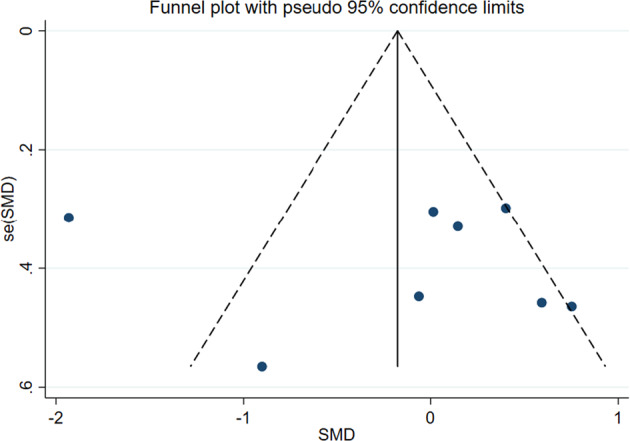
Funnel plot of HAMD scale score.

To evaluate whether there is publication bias in the included trials of MST, a funnel chart of HAMD scale score was used (Fig. 6). The funnel plot showed asymmetry, then Begg’s rank correlation and Egger’s regression test were computed to quantify the possible amount of bias, these tests remained non-significant (*P* = 0.805 and *P* = 0.662, respectively) (eFigs. 4 and 5). In summary, these complementary analyses support the absence of publication bias in this meta-analysis.

## Discussion

This study is the first systematic review and meta-analysis of the clinical efficacy of MST on antidepressant treatment and its impact on cognitive function. In terms of antidepressant efficacy, there was no significant difference in HAMD scores between the MST and ECT groups at baseline and post-treatment levels, but there was a significant decrease in HAMD scores compared with baseline levels, confirming that the efficacy of MST and ECT antidepressant treatment is equivalent; in terms of reorientation and recovery time, the two groups of reorientation time and recovery time are statistically significant. And the reorientation time and recovery time of MST group were shorter than that of the ECT group, which confirmed that the side effect of MST is less than ECT; in terms of each cognitive dimension, the MST group scored significantly higher than the ECT group in words immediate recall, word delayed recall, visual-spatial immediate recall, visual-spatial delayed recall, and verbal fluency tests, which confirmed that MST treatment had less impact on cognitive function than ECT treatment. In conclusion, this study provides the first evidence-based medicine support for the efficacy and fewer side effects of MST antidepressant treatment.

This study confirmed that MST has the same antidepressant efficacy as ECT, which was consistent with the initial findings of Hoy et al. [33]. Hoy et al. first studied the neural mechanism of MST influence and found the influence of MST on depression-related brain areas [34, 35]. Kayser et al. also found that MST is related to local metabolic changes in depression-related brain regions, and induces neuroplasticity in the frontal cortex through a long-term potentiation-like mechanism, thereby reducing suicidal ideation in patients with depression, which confirmed the antidepressant effect of MST from the perspective of neural mechanism [11, 36]. The above conclusion is slightly different from Fitzgerald [28], who reported two factors that might explain a lower remission rate of MST treatment. One is that the sample size of the study is small, and it is not a controlled study with ECT; confounding factors such as the length of treatment in different studies and the location of stimulation may impact the consistency of the results. This study included all the controlled trials that have used MST and ECT antidepressant treatments so far and balanced the above confounding factors. Given the novelty of MST technology, current research data is still limited. Therefore, more detailed clinical studies are needed to determine the best parameters of MST matched with different mental diseases.

Another major finding of this study is that in antidepressant treatment, MST has less impact on cognitive impairment than ECT. Moreover, several studies showed that MST has a significant positive impact on cognitive function in patients with depression. The first research applied MST in the treatment of depression patients published in 2001, which had confirmed the feasibility and safety of MST [7]. In this study, patients received four treatments to improve their mood and got few cognitive side effects. Besides, Lisanby et al. studied the neural mechanism of MST treatment and found that the magnetic stimulation of MST did not reach the hippocampus, which means that it may have no side effects on memory. On the contrary, MST can promote memory improvement [5, 16]; Wang et al. also found that MST has a significant effect on memory improvement. Besides, Kayser et al. also found that compared with ECT, MST has a positive effect on the cognitive function in depression [14, 28, 29]. Future research needs to be conducted to explore the detailed impact of MST on patients’ different cognitive dimensions, not only on memory but also a full view of MST on the cognitive effect.

This study found that compared with ECT, MST had a shorter reorientation time and recovery time after treatment. It may be related to the use of anesthetics. Due to the difference from other anesthetics used in Whit et al. studies [30], there was large heterogeneity in the study of recovery time. In addition, compared with ECT, MST stimulation did not affect the deep brain area, and the deep brain structure was less damaged, which may also explain the reason for the rapid recovery of consciousness in MST group. White et al. found that the difference in mean arterial blood pressure after stimulation may be related to low-dose calcium channel blocker nicardipine and the rapid recovery of cognitive function in MST group and ECT group [30]. Future clinical studies using MST should try to determine the optimal stimulus parameters, dose requirements, and predictors of a favorable response to treatment.

Heterogeneity exists in the 10 studies included in this study, which can be explained by several factors. Firstly, the course of treatment varies from 5 to 24 times, and El-Deeb et al. study may also result in significant differences [32]; Secondly, due to the differences caused by different anesthetics, the vast majority used propofol to induce anesthesia, succinylcholine to induce muscle relaxation, and a few used etomidate or atropine to induce anesthesia, resulting in different side effects. White et al.’s study may also have great differences [30]. Finally, the stimulus parameters used (the stimulus location, duration, and procedures) vary greatly. Besides, no adverse effects were found in the study included in the meta-analysis.

There are also some limitations in our study. Firstly, due to the limited reports of studies on both MST and ECT, the sample size is relatively small. Therefore, although the meta-analysis was used to further expand the sample size in this study, it still needs to be enriched. Secondly, the quality of the literature in this study is medium, and only two of them used the blind method with certain risks. The reason is that the current clinical trials of MST require the signing of informed consent. It is impossible to completely double-blind the subject and therapist. Therefore, it is only blinded by the evaluator, and there may be certain information bias. Thirdly, the included literature only evaluated the impact of short-term cognitive function but not long-term follow-up studies. Therefore, the impact of long-term cognitive function needs to be explored in the future. Due to the limitations of sample size, randomization, no longitudinal follow-up studies, and drug control, this study is only a preliminary evidence-based medical exploration of the efficacy and cognitive impact of MST in the treatment of depression. Despite a moderate effect for MST on the efficacy and cognition function of depression, larger-sample, long-term follow-up, and high-quality reports on MST are needed in the future.

## Conclusion

Our analyses indicate that MST may be an efficacious treatment for depression with an effect size similar to ECT. Besides, the impairment of cognitive function is significantly less than ECT. This study provides evidence for the efficacy and cognitive side effects of MST in antidepressant treatment. Therefore, future research on the effect of MST on cognitive function in the treatment of depression may be of great value, and MST is expected to become the preferred choice for antidepressant physical therapy.

## Supplementary information

supplementary materials
