# Supplementary material for: Comparative efficacy and cognitive function of magnetic seizure therapy vs. electroconvulsive therapy for major depressive disorder: a systematic review and meta-analysis
Source: Transl Psychiatry. 2021 Aug 21;11:437. doi: 10.1038/s41398-021-01560-y (PMC8380249; doi:10.1038/s41398-021-01560-y)
Supplement: Supplementary file 1 — supplementary materials [file 41398_2021_1560_MOESM1_ESM.docx]

**supplementary materials**

**eFigure 1:** Risk bias assessment of 10 studies

**eFigure 2:**Forest plot of the efficacy of depression symptoms in the MST group and ECT group in the treatment of depression after a literature removal

**eFigure 3:**Forest plot of the reorientation time and recovery time in the MST group and ECT group in the treatment of depression after literature removal

**eFigure 4:**Publication bias test of included articles

**eFigure 5:**Funnel plot of neuropsychological and Bias test of included articles

**eFigure 1:**


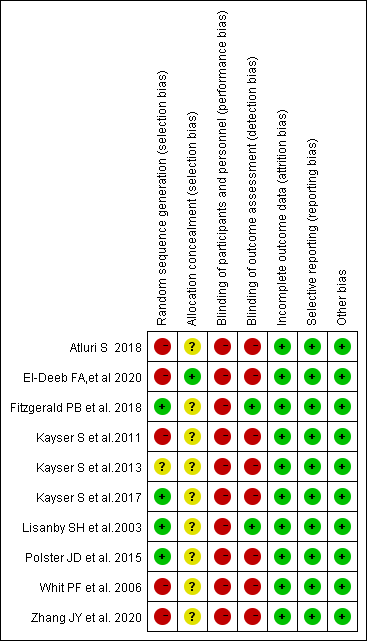


**Risk bias assessment of 10 studies**

**eFigure 2:**


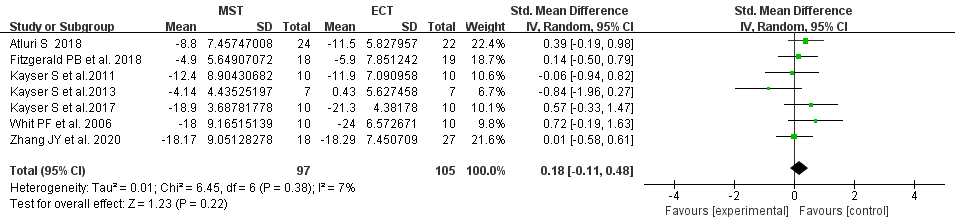


Forest plot of the efficacy of depression symptoms in the MST group and ECT group in the treatment of depression after a literature removal: Q = 6.45, *P =* 0.38*,* I^2^ = 7%.

**eFigure 3:**

**
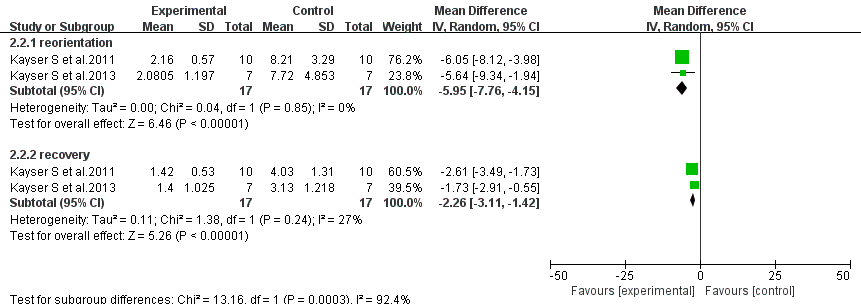
**

Forest plot of the reorientation time and recovery time in the MST group and ECT group in the treatment of depression after literature removal:Q = 1.38, *P =* 0.24*,* I^2^ = 27%.

**eFigure 4:**


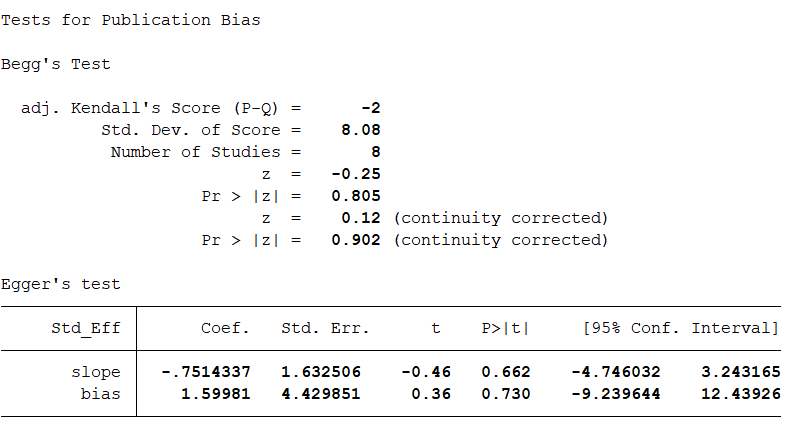


Publication bias test of included articles

**eFigure 5:**


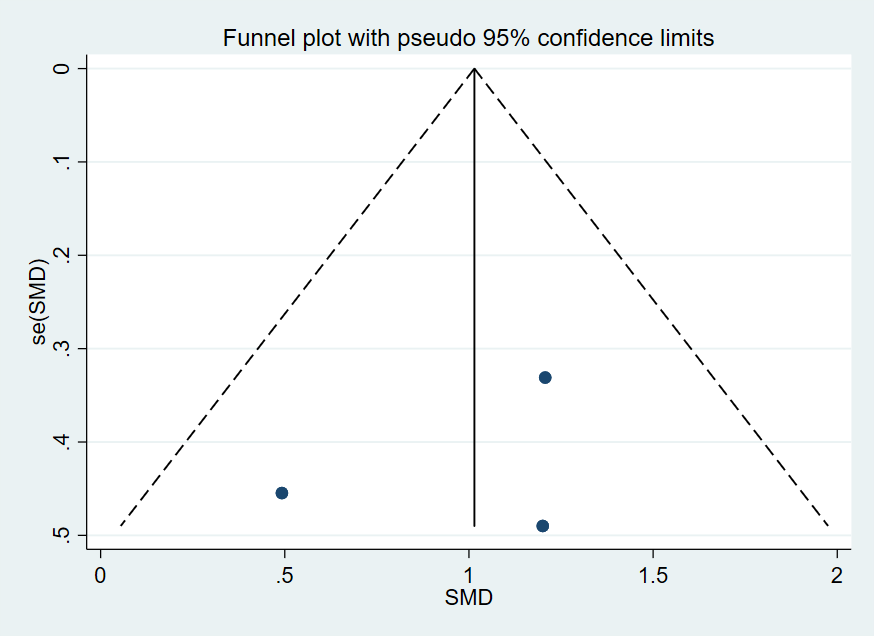


Funnel plot of word delayed recall score


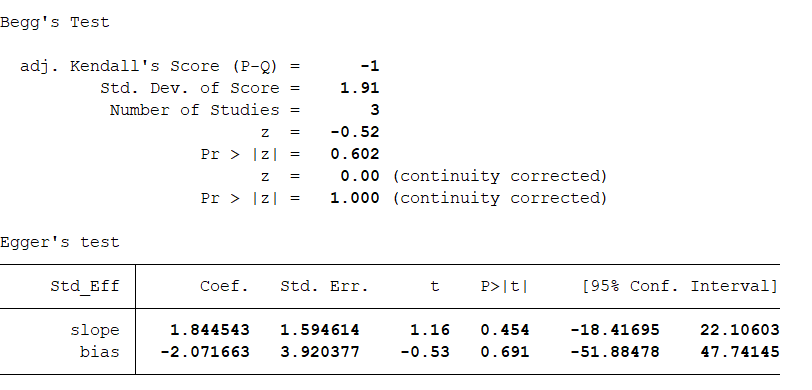


Bias test of 3 included articles

Although the number of included studies is relatively small, it is still found that the scattered points included in the research effect are evenly distributed, indicating that there is no obvious publication bias in the meta-analysis of this outcome indicator. At the same time, the bias test showed that p values were greater than 0.05, so it can be judged that there is no publication bias in this study.


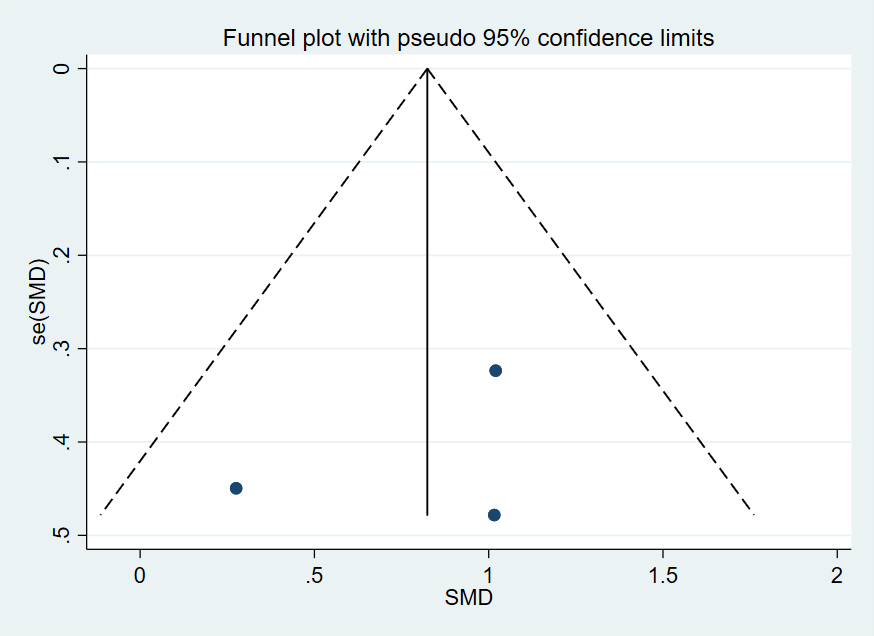


Funnel plot of word immediate recall score


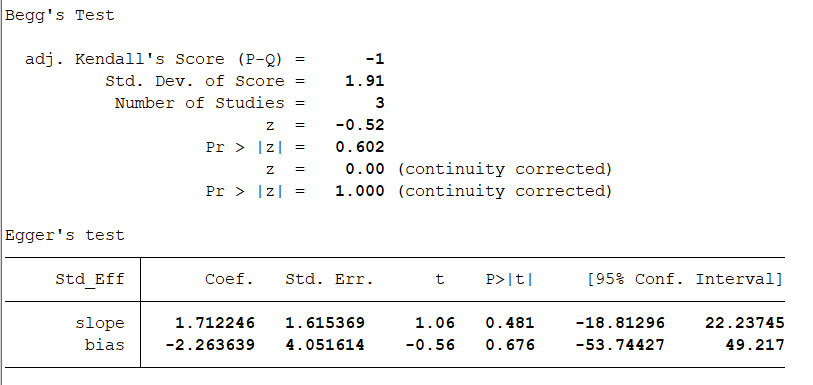


Bias test of 3 included articles

Although the number of included studies is relatively small, it is still found that the scattered points included in the research effect are evenly distributed, indicating that there is no obvious publication bias in the meta-analysis of this outcome indicator. At the same time, the bias test showed that p values were greater than 0.05, so it can be judged that there is no publication bias in the literature of this study.


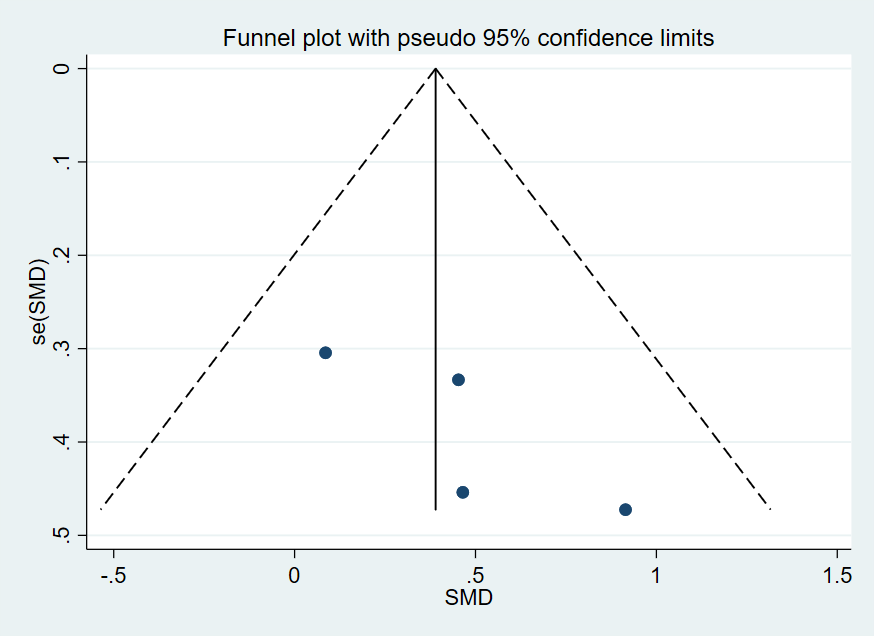


funnel plot of visual spatial immediate memory score


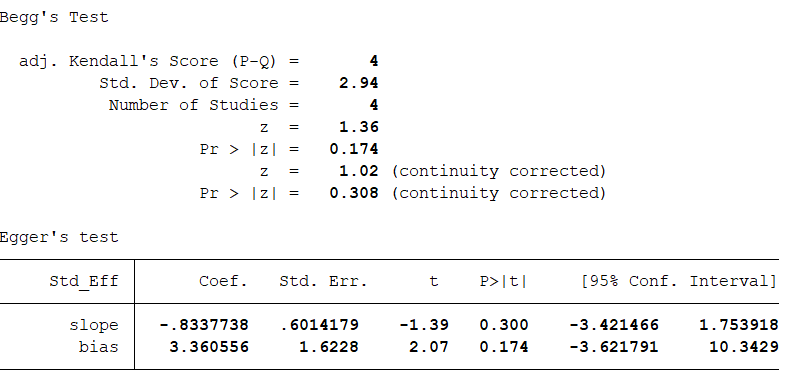


Bias test of 4 included articles

Although the number of included studies is relatively small, it is still found that the scattered points included in the research effect are evenly distributed, indicating that there is no obvious publication bias in the meta-analysis of this outcome indicator. At the same time, the bias test showed that p values were greater than 0.05, so it can be judged that there is no publication bias in this study.


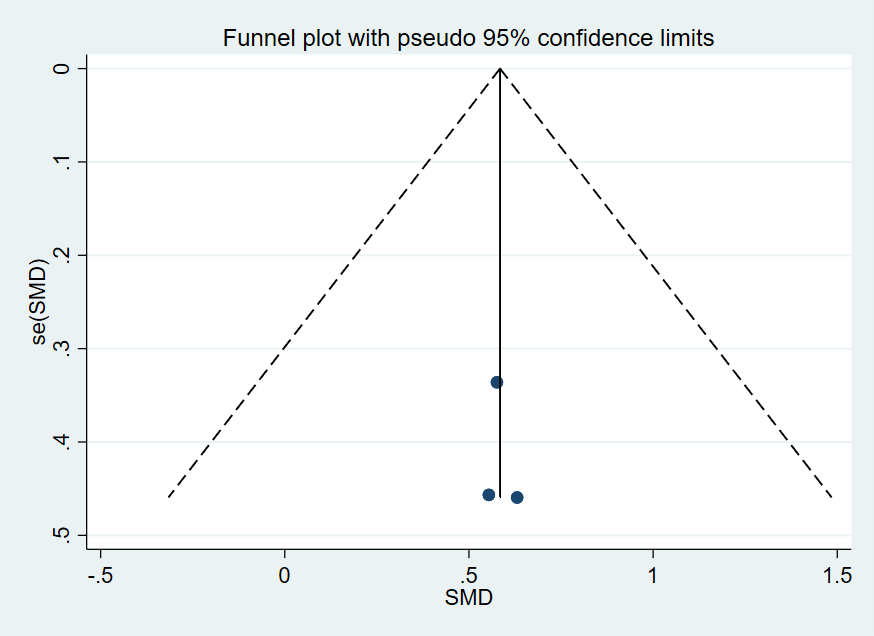


Meta-analysis funnel plot of visual spatial delayed recall


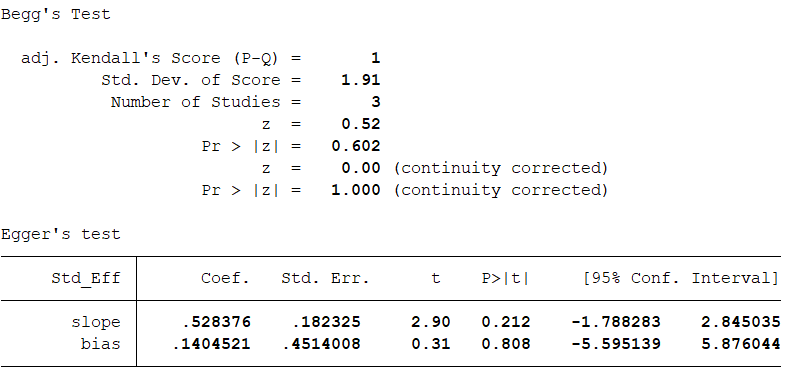


Bias test of 3 included articles

Although the number of included studies was small, the scattered points of the included studies were still evenly distributed and arranged around the central line, indicating that there was no significant publication bias in the meta-analysis of the outcome index. At the same time, the bias test showed that p values were greater than 0.05, so it can be judged that there is no publication bias in this study.


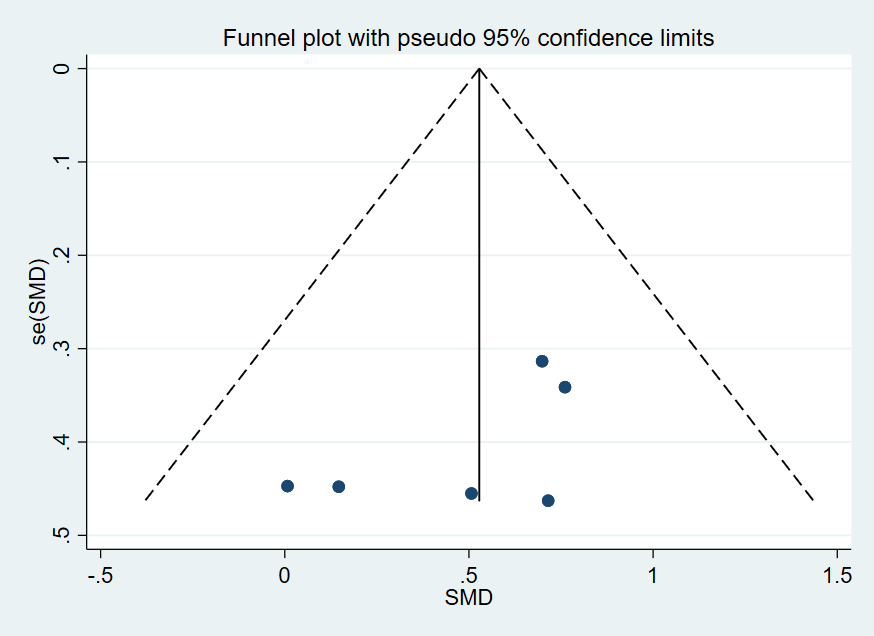


funnel plot of language fluency


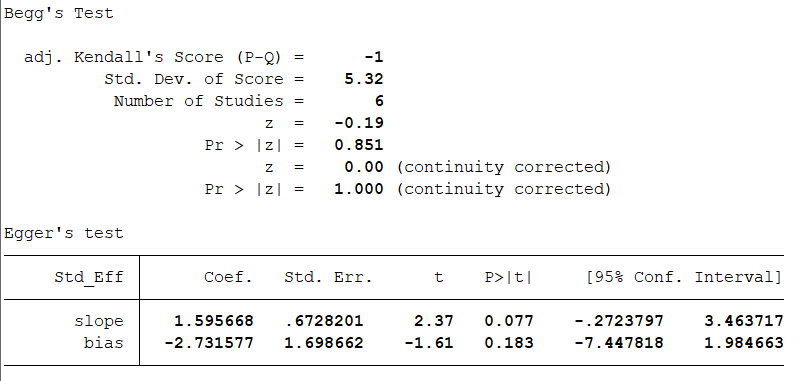


Bias test of 3 included articles

Although the number of included studies is relatively small, it is still found that the scattered points included in the research effect are evenly distributed, indicating that there is no obvious publication bias in the meta-analysis of this outcome indicator. At the same time, the bias test showed that p values were greater than 0.05, so it can be judged that there is no publication bias in this study.
